# Fifteen millennia of human mitogenome evolution in Sicily

**DOI:** 10.1126/sciadv.ady1674

**Published:** 2025-11-14

**Authors:** Anna Tommasi, Rajiv Boscolo Agostini, Giacomo Villani, Nicola Rambaldi Migliore, Maria T. Vizzari, Irene Cardinali, Rosalinda Di Gerlando, Valeria Nicolini, Gary Sorasio, Patrícia Santos, Anna Olivieri, Ugo A. Perego, Giulio Catalano, Nicoletta Volante, Lucia Sarti, David Caramelli, Luca Sineo, Hovirag Lancioni, Alessandra Modi, Silvia Ghirotto, Alessandro Achilli

**Affiliations:** ^1^ Department of Biology and Biotechnology “L. Spallanzani”, University of Pavia, Pavia 27100, Italy.; ^2^ Department of Life Sciences and Biotechnology, University of Ferrara, Ferrara 44121, Italy.; ^3^ Department of Chemistry, Biology and Biotechnology, University of Perugia, Perugia 06123, Italy.; ^4^ IRCCS Mondino Foundation, Pavia 27100, Italy.; ^5^ Southeastern Community College, West Burlington, IA 52655, USA.; ^6^ Department of Biological, Chemical and Pharmaceutical Sciences and Technologies, University of Palermo, Palermo 90123, Italy.; ^7^ Department of History and Cultural Heritage, University of Siena, Siena 53100, Italy.; ^8^ Department of Biology, University of Florence, Florence 50122, Italy.

## Abstract

Sicily, situated at the heart of the Mediterranean Sea, has been a crossroads of people of different origins since the Paleolithic. To gain further insight into the genetic history of this island from a matrilineal viewpoint, we investigated 15 millennia of human mitogenome evolution. A unique Sicilian mitochondrial DNA (mtDNA) dataset, represented by 116 ancient mitogenomes (including two newly sequenced) collected from 16 archeological sites dating from 14,700 to 545 years ago, was compared with a collection of 236 modern mitogenomes covering all districts of the island. By integrating demographic modeling with phylogeographic analyses, we identified a statistically supported genetic discontinuity between the Paleolithic/Late Mesolithic and Early Neolithic periods and two mtDNA lineages (U5b and U8b/K) that specifically mark this transition. The extensive variation and lack of genetic structure among modern mitogenomes suggest the presence of a continuous, maternally inherited gene flow from different regions of Western Eurasia (since the Paleolithic) and Africa (since the Bronze Age).

## Teaser

Mitogenomes spanning 15 millennia reveal a genetic discontinuity within Sicily and continuous gene flow from Eurasia and Africa.

## INTRODUCTION

Located at the center of the Mediterranean Sea, Sicily has long served as a pivotal crossroads for human migrations. Since its initial colonization, possibly during the Late Upper Paleolithic (*1*), the island has been settled by a wide range of populations, including Neolithic farmers from Anatolia and the Near East, Italic groups from the mainland, Phoenicians, Sicani, Greeks, Romans, Byzantines, Arabs, and Normans (*2*). This complex and continuous interplay of demic and cultural dynamics has deeply influenced the biological and cultural landscape of Sicily (*3*). However, the extent to which these historical processes have shaped the genetic structure of the island’s populations, both through time and in the present, is still poorly understood.

Previous studies on modern Sicilian genetic variation have focused on uniparental markers. Analyses of mitochondrial DNA (mtDNA) control regions (mostly hypervariable sequences I and II) and Y-chromosome polymorphisms have suggested a relatively homogeneous genetic landscape across the island (*4*–*6*). However, these studies were limited by sample size and lacked high-resolution data. To date, no complete mitochondrial genomes from modern Sicilian individuals have been published, resulting in an incomplete phylogenetic resolution of maternal lineages.

Autosomal data have provided broader insights: Sarno *et al.* (*7*) analyzed modern individuals from six Sicilian municipalities and revealed a strong genetic affinity with southern Italy and the eastern Mediterranean, including a predominant Neolithic component alongside contributions from post-Neolithic Caucasian and Levantine ancestries. However, in the absence of ancient genetic data, it remains unclear when these ancestries arrived on the island, which populations contributed to them, and how these genetic components evolved over time.

Ancient DNA studies have begun to fill this gap, although data remain sparse. Early research focused on the initial colonization of the island and revealed low genetic diversity among Upper Paleolithic hunter-gatherers, possibly due to genetic drift (*8*, *9*). Genome-wide data from the Late Epigravettian individual Oriente C confirmed a western hunter-gatherer (WHG) ancestry and suggested relative homogeneity among Epigravettian populations in the central Mediterranean (*10*, *11*). More recently, Fernandes *et al.* (*12*) analyzed genome-wide single-nucleotide polymorphisms (SNPs) from 24 ancient individuals dating from the Middle Neolithic to the Late Bronze Age, detecting Steppe-related gene flow during the Bronze Age. Modi *et al.* (*13*) generated complete mitochondrial genomes from 36 individuals spanning the Bronze Age to the Hellenistic period, revealing a structured maternal genetic variation in Sicily since the Early Bronze Age and supporting a demic impact of cultural transitions. However, this study lacked a comprehensive chronological review and a comparison with modern mitogenomes, as previously performed on Sardinia, the other major Mediterranean island (*14*). To date, only broad haplogroup assignments have been available for contemporary Sicilians, preventing fine-scale phylogenetic or temporal inferences.

In this study, we present the first complete mitochondrial genomes from modern Sicilian populations. We sequenced the complete mitogenome of 236 individuals from all 9 provinces, covering the full geographic extent of the island. We also assembled a comparative dataset of 116 ancient complete mitochondrial genomes spanning from the Paleolithic to the Medieval period, including two newly generated sequences from a Late Mesolithic individual from Grotta dell’Uzzo (Trapani) and an Imperial-period individual from Grotta di San Teodoro (Messina).

By analyzing this extensive dataset, we aimed to (i) reconstruct the phylogenetic history and temporal dynamics of maternal lineages in Sicily, (ii) assess the presence and persistence of genetic structure through time, and (iii) quantify maternal genetic continuity since the island’s initial colonization while evaluating the impact of successive migration events and cultural transformations.

## RESULTS

**Fig. 1.**
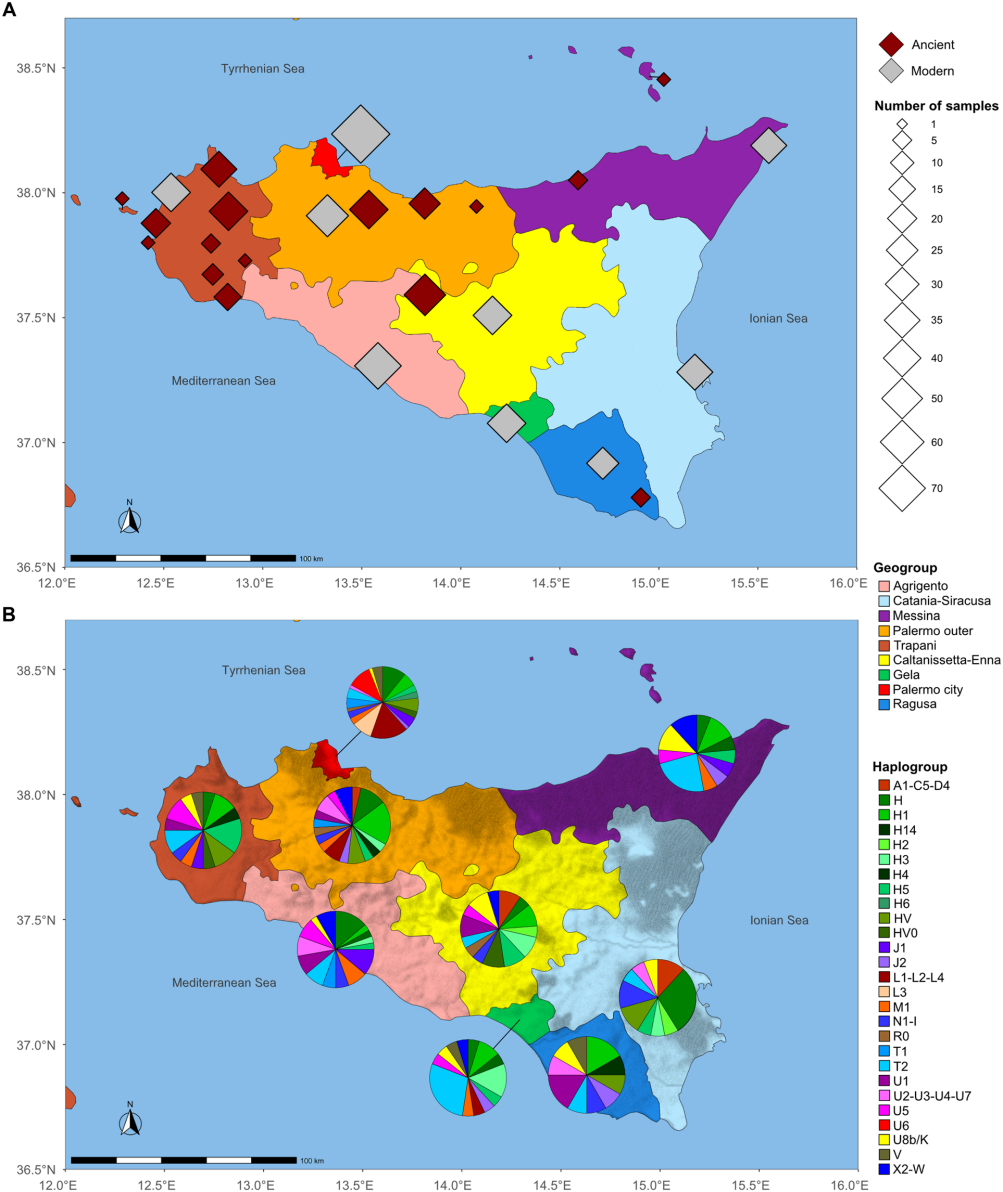
Origin of mitogenomes and haplogroup frequencies. (**A**) Geographic origin of ancient and modern mitogenomes analyzed in this study and (**B**) haplogroup distribution in present-day Sicily. Silver diamonds mark the geographic origin of the terminal maternal ancestor (TMA) from modern individuals (*N* = 236), distributed in nine established geogroups highlighted in different colors. Brown diamonds indicate the archeological sites where the ancient samples (*N* = 116) were excavated. See datasets S1, S2, and S3A for further details.

Sicily is one of the largest and most centrally located islands in the Mediterranean Sea, connecting Africa and Europe. Hence, it has always been a melting pot for human migrations between Europe and the Mediterranean basin, becoming the perfect environment for complex biological and cultural population dynamics (*4*, *15*). To characterize its genomic diversity, we focused on the mtDNA. Two newly generated ancient mitogenomes were combined with 114 previously published ancient mitogenomes spanning several time periods (from the Paleolithic to the Middle Ages) and compared with a modern dataset of 236 mitogenomes, fully sequenced for the purpose of this study to represent the whole mitochondrial variation of present-day Sicily (Fig. 1A and datasets S1 and S2). One of the novel ancient mitogenomes belonged to a Mesolithic individual from the Grotta dell’Uzzo archeological site in Trapani, while the other was derived from a more recent individual dated to the Roman period from the Grotta di San Teodoro archeological site in Messina.

### mtDNA variation of present-day Sicily

The mitochondrial variation of present-day Sicily was initially evaluated in detail through a phylogenetic analysis of 236 high-quality modern mitogenomes. This analysis was conducted after the island was divided into nine “geogroups” based on political provinces, sample sizes, and orography. A high level of mtDNA variability is evidenced by 226 haplotypes, with a high diversity index (Hd = 0.99), and 175 haplogroups and subhaplogroups (dataset S1). The haplogroup origin has been derived from various papers on mtDNA phylogeography (*14*, *16*–*18*). The majority of lineages have an origin from Western Eurasia, representing more than 80% of the total. Approximately 9% have a North African origin, while around 6% can be traced back to sub-Saharan Africa. It is noteworthy that a small proportion of the identified haplogroups (2%) have their origin in East Asia (Fig. 1B and datasets S1 and S3A). The differential distribution of 27 macrohaplogroups among the nine geogroups was evaluated using correspondence analysis (CA) (fig. S1A), which did not yield any evidence of genetic structure. The distinctive presence of mitochondrial lineages of African origin—specifically haplogroups L1, L2, L3, L4, M1, and U6—accounts for 40% of the genetic composition of Palermo city and is the primary factor contributing to its outlier position. This pattern was also confirmed after the number of individuals from Palermo city was reduced from 65 to 30 to obtain a sample size comparable to that of the other geographic groups (fig. S1B). Even when Palermo city was excluded from the CA (fig. S1C), no discernible structure could be identified.

The lack of genetic structure in the Sicilian population was validated through a direct comparison of various demographic models (figs. S2 to S4). In this analysis, the modern individuals were divided into distinct subgroups based on geographic proximity (fig. S4). Simulated genetic variability under two alternative demographic models, representing no modern genetic structure and an east-west genetic separation, were compared through an Approximate Bayesian Computation (ABC) framework. This analysis suggests the absence of population structure among contemporary Sicilians (see also the section “Evolution of the mitochondrial gene pool of Sicily across 15 millennia”). Accordingly, the modern mitogenome dataset was treated as a single entity in subsequent analyses to be compared with the ancient mitogenomes from disparate time periods.

### Evolution of the mitochondrial gene pool of Sicily across 15 millennia

The ancient individuals were divided by their archeological sites and time transects, with consideration given to the various cultures represented: Phoenicians/Punics in Motya, Greeks and Sicanians in Baucina, Christian, and Islamic burials in Segesta.

**Fig. 2.**
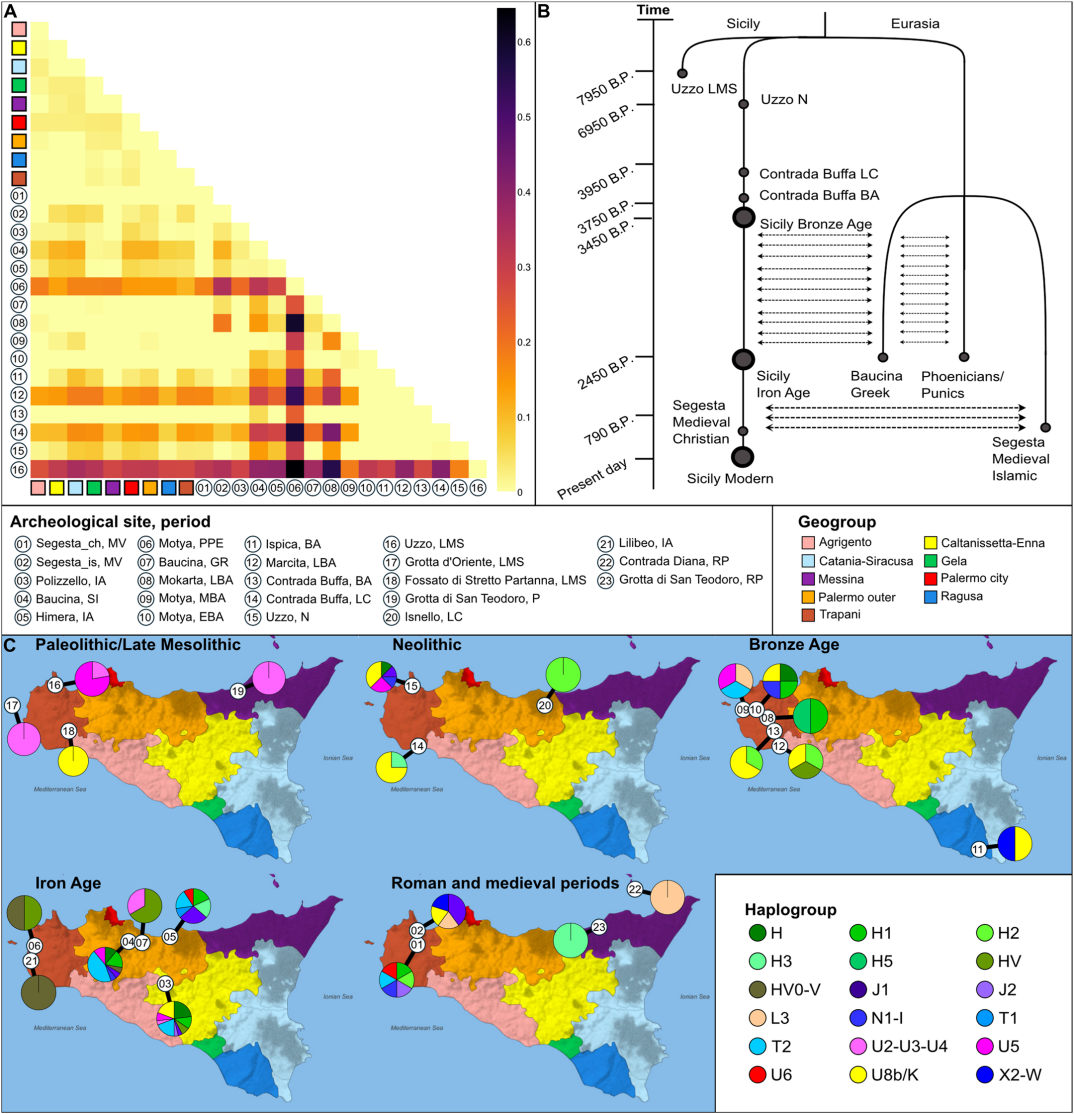
Exploratory analyses of the mtDNA genetic diversity over time. (**A**) Heatmap representation of *F*_ST_ values computed between groups of individuals. Legend in the central panel. (**B**) Best demographic model, describing the observed genetic discontinuity between the Uzzo Mesolithic group and Uzzo Neolithic. (**C**) Haplogroup frequencies in ancient Sicily; see dataset S3B for further details. Legend in the central panel as in (A) and in the bottom right panel, with each color representing a geographic location, while each number indicating a time transect/culture. Cultures in the legend are labeled as follow: P, Paleolithic; LMS, Late Mesolithic; N, Neolithic; LC, Late Chalcolithic; BA, Bronze Age; EBA, Early Bronze Age; LBA, Late Bronze Age; IA, Iron Age; PPE, Phoenicians/Punics; SI, Sicanians; GR, Greeks; and RP, Roman Period.

The initial exploratory analyses of genetic diversity over time were conducted through the computation of the fixation index (*F*_ST_) between pairs of populations (*19*). The results indicate a low level of genetic differentiation (*F*_ST_ < 0.06) in modern populations. In contrast, ancient populations exhibit higher values, with Late Mesolithic individuals from Grotta dell’Uzzo and Phoenicians/Punics from the island of Motya representing the most differentiated populations, displaying *F*_ST_ values ranging from 0.2 to 0.6 (Fig. 2A).

Populations from the Late Iron/Classical Age—including those from Himera, Baucina, and Polizzello—exhibit greater similarities to modern populations (*F*_ST_ < 0.06). The *F*_ST_ values observed in medieval individuals from the Islamic cemetery in Segesta are comparable to those seen in Iron Age populations. Conversely, individuals from the Christian cemetery exhibit no differentiation from modern populations (*F*_ST_ = 0). To facilitate visualization of these patterns, a multidimensional scaling was performed on the *F*_ST_ matrix (fig. S1D). It is evident that Phoenicians/Punics from the island of Motya and Islamic individuals from Segesta are distinctly separated from all other groups. This is also the case, to a lesser extent, with Late Mesolithic individuals from Grotta dell’Uzzo and Chalcolithic individuals from Contrada Buffa. Some modern groups from Palermo, Gela, Caltanissetta, and Messina are more closely related to Bronze Age and Iron Age individuals, suggesting a potential genetic continuity since the Bronze Age.

The aforementioned genetic patterns are explicable within the context of a range of evolutionary hypotheses. To investigate the evolutionary history of Sicily from the Mesolithic to the modern era, we used demographic modeling through ABC. In consideration of both chronology and the genetic affinities derived from previous *F*_ST_ analyses, the analyzed individuals were chronologically grouped into 11 distinct groups of at least three individuals: the Mesolithic Grotta dell’Uzzo; the Neolithic Grotta dell’Uzzo; the Chalcolithic Contrada Buffa; the Bronze Age Contrada Buffa; all other Bronze Age individuals; the Iron Age; the Phoenicians/Punics from Motya; the group from Baucina, considered to be of Greek origin, although probably already mixed with local communities; the Islamic individuals from Segesta; the Christian individuals from Segesta; and all modern individuals.

A hierarchical ABC approach (*20*, *21*) was used to initially assess the five primary topologies describing divergences between groups without consideration of migration (fig. S2, A to E, and dataset S4). Model A, which accounts for complete genetic continuity over time, was identified as the optimal model with a posterior probability of 84.2% (dataset S5). A second model selection test was then performed, including known events of migration, such as those between Iron Age Sicily, Phoenicians/Punics, and mixed Greeks groups and between medieval Segesta groups (fig. S2, F to I, and dataset S4). In the second comparison, model G was identified as the most supported model, accounting for a discontinuity between the Mesolithic and Neolithic Sicilian populations, with a posterior probability of 70% (dataset S5). The final comparison was between model A and model G, with model G being selected with a posterior probability of 86.5%. These comparisons demonstrated minimal classification errors (*CE*s) and a low out-of-bag prior error rate (2.3, 5.5, and 0.7%, respectively), indicating substantial statistical support for these findings (dataset S5).

In summary, after evaluating nine demographic models, the most statistically supported model suggests a genetic discontinuity between the Mesolithic and Neolithic periods in ancient individuals from the Grotta dell’Uzzo site, followed by a genetic continuity in Sicily until the present day (Fig. 2B). The Greek and Phoenician/Punic populations are distinguished from the Sicilian groups and are typified by uninterrupted two-way migration patterns. Individuals of Islamic faith from Segesta are distinguished from those of Christian faith, who are in continuity with Sicilian groups, and are characterized by two-way continuous migration during the medieval period. Having inferred the most appropriate demographic model to represent the mitochondrial gene pool history in Sicily, we proceeded to investigate the presence of genetic structure between modern Sicilian populations, as previously mentioned. To perform this analysis, we considered the modern Sicilian population, which was structured into nine geographical groups. We designed two demographic variants of model G: Model Gpan, which is a panmictic model in which all modern groups are exchanging migrants at the same rate, and Model Gst, which is a structured model in which the western (Palermo city, Palermo outer, Trapani, and Agrigento) and eastern (Caltanissetta-Enna, Catania-Siracusa, Gela, Messina, and Ragusa) geographical groups are genetically separated. The ABC comparison indicated a 58.5% posterior probability for Model Gpan. However, this final model selection was characterized by high error rates (out-of-bag prior error rate of 39% and *CE* rates of 38% and 40%, respectively), thus indicating that any genetic structure is difficult to detect under our experimental conditions. Therefore, the evidence provided by our analyses is compatible with a panmictic model for the modern population, with no structured mtDNA variation (fig. S3D and dataset S6).

To gain further insight into the temporal dynamics of mitochondrial variation, we conducted an in-depth analysis of the frequencies of macrohaplogroups across five distinct historical periods. The historical periods under consideration are the Paleolithic (including the Early and Late Mesolithic), Neolithic, Bronze Age, Iron Age, and the Medieval/Roman period (Fig. 2C). Among the 12 Paleolithic individuals, dated between 15,950 and 7950 years before present (yr B.P.), 11 distinct haplotypes were identified, with a high diversity index (Hd = 0.98). During this period, three macrohaplogroups were identified: U2-U3-U4 (*n* = 4), U5 (*n* = 7), and U8b/K (*n* = 1). The U5 haplogroup was the most prevalent among the sequences from individuals at Grotta dell’Uzzo, representing 87% (seven of eight) of the total. In contrast, macrohaplogroup U2-U3-U4 was distributed across multiple sites, including Grotta dell’Uzzo, Grotta d’Oriente, and Grotta di San Teodoro, covering both the northern and western areas of Sicily. A single U8b/K mitogenome was identified at Fossato di Stretto Partanna. It is noteworthy that radiocarbon dating places this individual in the Neolithic, while the archeological context suggests a Late Mesolithic attribution (*12*). Overall, the genetic composition of the Sicilian population during the Paleolithic reflects a predominance of haplogroups linked to Western Eurasia.

In regard to the Neolithic period (7949 to 4450 yr B.P.), our analysis encompassed 13 mitogenomes, each exhibiting a distinct haplotype and representing eight macrohaplogroups from Western Eurasia. These include H (*n* = 1), H2 (*n* = 1), H3 (*n* = 1), J1 (*n* = 1), N1-I (*n* = 1), U8b/K (*n* = 6), and U5 (*n* = 2). It should be noted that the previously prevalent Paleolithic macrohaplogroup U2-U3-U4 is absent in the Neolithic, reflecting its earlier isolation in northwestern Sicily. In contrast, the emergence of new haplogroups—including H, H2, J1, and N1-I—indicates a substantial reorganization of the genetic landscape in the island. Moreover, the U8b/K haplogroup is particularly frequent, comprising 37% of the analyzed mitogenomes (three of eight) at Grotta dell’Uzzo and 75% (three of four) at Contrada Buffa. This underscores its prominence in the Neolithic populations of northwestern Sicily. These findings highlight a dynamic shift in mitochondrial diversity, which is likely reflective of broader cultural and demographic changes that occurred during this period.

The Bronze Age (4449 to 2900 yr B.P.) mitogenome dataset comprises 17 sequences, which reveal 16 distinct haplotypes with a large haplotype diversity (Hd) of 0.99. The sequences were assigned to 11 macrohaplogroups and 15 subhaplogroups. New haplogroups were introduced into the Sicilian gene pool, including H (*n* = 1), H1 (*n* = 2), H2 (*n* = 2), H5 (*n* = 1), HV (*n* = 1), T2 (*n* = 1), and X2 (*n* = 1). With the notably exception of L3 (*n* = 1), which is associated with North Africa, all of these are of Western Eurasian origin. It is noteworthy that U8b/K emerged as the most prevalent and widely distributed haplogroup, with a frequency of 66.7% (two of three) at Marcita in northwestern Sicily and 50% (one of two) at Ispica in the south/east. The highest genetic variability during the Bronze Age was observed at the archeological site of Motya, where the presence of the L3 lineage suggests a potential genetic link between Sicily and the African continent.

The Iron Age (2899 to 2550 yr B.P.) was a period of crucial changes, as evidenced by the appearance of three distinct civilizations in written chronicles: the Elymians, the Sicanians, and the Sicels (*22*–*24*). Each of the 61 mitochondrial genomes from this period exhibited a distinct haplotype, indicating notable shifts in haplogroup frequencies, including an increase in T2 (*n* = 15) and HV (*n* = 5). Among these, six individuals associated with the Sicanians from Baucina were assigned to haplogroup T2, while two Greek individuals from the same site belonged to haplogroup HV. The archeological sites of Motya and Lilibeo yielded evidence of haplogroup HV0-V (*n* = 2), while the identification of a U6 lineage (*n* = 1) associated with North Africa at the archeological site of Himera underscores the sustained genetic exchanges with the African continent.

In the Roman period (2549 to 1400 yr B.P.), two individuals were analyzed: one with the L3 lineage from the Contrada Diana site (situated on Lipari Island) and one with the H3 lineage from Grotta di San Teodoro. During the Medieval period (1399 to 458 yr B.P.), 11 mitochondrial genomes were identified at the archeological site of Segesta, which featured two distinct cemeteries: one Christian and one Islamic. Among the individuals interred in the Islamic cemetery, four were found to belong to Western Eurasian haplogroups, specifically U8b/K (*n* = 1), J1 (*n* = 2), and X2-W (*n* = 1). One individual, however, exhibited the L3 lineage. In the Christian cemetery, five individuals were identified as belonging to Western Eurasian haplogroups, specifically T2 (*n* = 1), H2 (*n* = 1), J2 (*n* = 1), H1 (*n* = 1), and N1-I (*n* = 1). In addition, one individual was found to carry the U6 lineage.

### A phylogenetic focus on haplogroups U5b and U8b/K

**Fig. 3.**
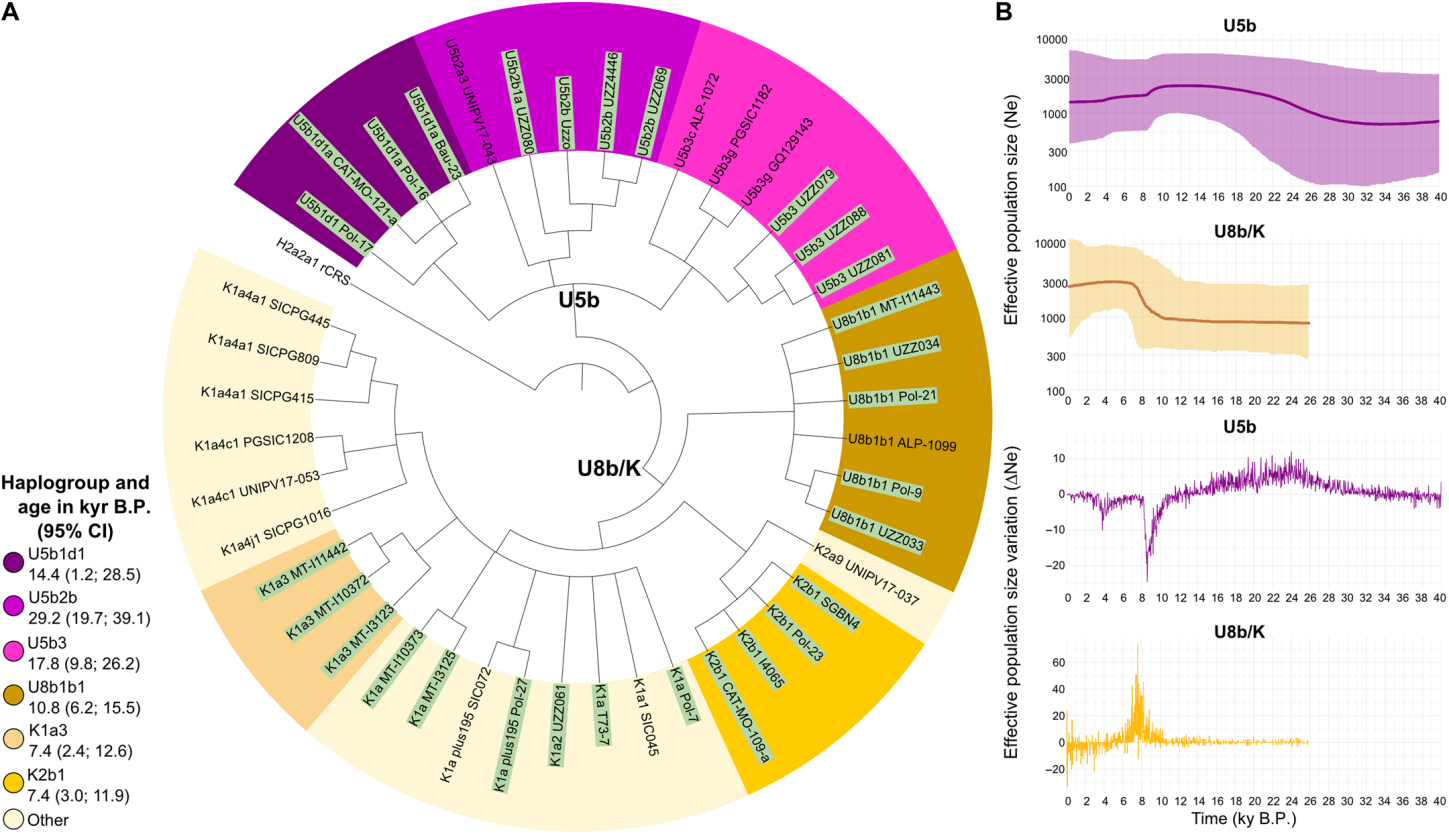
Phylogenetic tree and demographic trends of U5b and U8b/K. (**A**) MP tree encompassing 29 ancient and 14 modern Sicilian mitogenomes. Ancient samples are highlighted in light green. Name and age of the main branches are reported in the legend; kyr, thousand years; CI, confidence interval. See datasets S7 to S9 for further details. (**B**) BSPs and relative change of the effective population size (Ne) over time computed in 1500 time windows.

A total of six of the eleven identified Paleolithic lineages in Sicily belong to the haplogroup U5b, while approximately half of the Neolithic mitogenomes are U8b and K (six of 13). U5b has been identified as a marker for hunter-gatherers (*9*, *25*, *26*), whereas U8b, which encompasses K, is known for its spread in continental Europe and the Mediterranean basin with the advent of the Neolithic (*27*). To elucidate further the emergence and dispersal of these maternal lineages in Sicily, we constructed a maximum parsimony (MP) phylogenetic tree incorporating ancient and modern mitogenomes representative of the two maternal lineages (15 U5b and 28 U8b mtDNAs; Fig. 3A). The sub-branches U5b1d1 and U5b2b are exclusive to ancient individuals within the U5b lineage, whereas U5b3 is present in ancient and/or modern individuals. With regard to U8b, all Sicilian mitogenomes are classified as U8b1b1 (one modern and five ancient mtDNAs) and K (nine modern and 13 ancient ones). The former is extremely rare in contemporary Europe (less than 0.1%), whereas the latter is pervasively distributed (exceeding 5%) among the records in accessible databases. Within the K haplogroup, the sub-branches K2b1 and K1a3 are exclusive to ancient mitogenomes, whereas K1a4 appears solely in modern individuals.

The age of these haplogroups in Sicily was estimated by computing the mean molecular divergence within each branch and converting this value into time according to the current mitogenome molecular clock (*28*). Moreover, the average estimates were reevaluated by incorporating the hypothetical number of mutations that the ancient haplotypes could have accumulated up to the present day (recalibrated-rho dating, see Materials and Methods). This novel approach resulted in the backdating of all the analyzed haplogroups, which largely overlap with the 95% highest posterior density of the Bayesian estimates (dataset S7).

A focus on the primary sub-branches encompassing ancient Sicilian mitogenomes revealed that the three principal U5b branches (U5b1d1, U5b2b, and U5b3) are all dated to pre-Neolithic times, indicating an early presence of these haplogroups in the island since the Paleolithic and Mesolithic periods. In contrast, the U8b/K sub-branches (U8b1b1, K1a3, and K2b1) are dated to the Neolithic period (Fig. 3A). These different trends are evident in demographic Bayesian skyline plots (BSPs; Fig. 3B). The Sicilian U5b mtDNAs show an expansion from more than 30 thousand years (kyr) B.P. to the onset of the Last Glacial Maximum (LGM; 25 to 15 kyr B.P.) and a decline in the Early Neolithic, perfectly overlapping with the contemporary burst in population size represented by the Sicilian U8b/K mtDNAs (Fig. 3B). These results suggest a certain degree of population replacement on the island, as previously suggested by the analysis of dietary changes and genetic variability (*29*). In our study, we were able to identify U5b1d1, U5b2b, and U5b3 as mitochondrial markers of Paleolithic/Mesolithic Sicily and U8b1b1, K1a3, and K2b1 as characteristic of Neolithic population incomers.

### Searching for the origin of ancient mitochondrial footprints in Sicily

To gain further insights into the history of these newly identified mitochondrial markers of Paleolithic/Mesolithic and Neolithic Sicily, two comparative datasets containing all the currently published ancient and modern mtDNAs belonging to the selected haplogroups were created and reclassified using Haplogrep 3.2.1 (datasets S10, A and B) (*30*). Subsequently, we investigated the geographic and/or population origins of the identified mitogenomes, mapping archeological sites or, in the case of modern sequences, the capitals of the countries where the individuals were collected. The final aim was to map specific mitogenome signals related to different time periods, as shown in the movie S1 and discussed in the following section.

### Paleolithic/Mesolithic signals

The U5b1d1 branch was present in Germany and predominantly in Serbia during the Paleolithic era. This observation, in conjunction with its estimated age of 14,389 yr B.P., may suggest an exit from the Balkan refuge. During the Neolithic period, this lineage expanded into Central Europe and Spain, arriving in Sicily during the Bronze Age, specifically in Motya, where it remained through the transition from Iron Age to Classic Age. It was also present at Baucina Monte-Falcone and local sites. The current distribution of this haplogroup includes Europe and Egypt. However, it remains a rare haplogroup, accounting for only 0.04% of the total in online available datasets (*31*–*33*).

Another Paleolithic haplogroup, U5b2b, dated to 29,218 yr B.P., was already widely distributed during the Paleolithic throughout southern and eastern Europe, from the Iberian Peninsula to present-day Ukraine, with a notable presence at Grotta dell’Uzzo in Sicily. This distribution appears to mirror the ice line during the LGM, suggesting multiple expansions from all glacial refuges, including the Ukrainian one, at least from a mitochondrial (female) perspective. In subsequent periods, this haplogroup spread across Western Eurasia and Central Asia, maintaining this distribution up to the present day (0.5%).

**Fig. 4.**
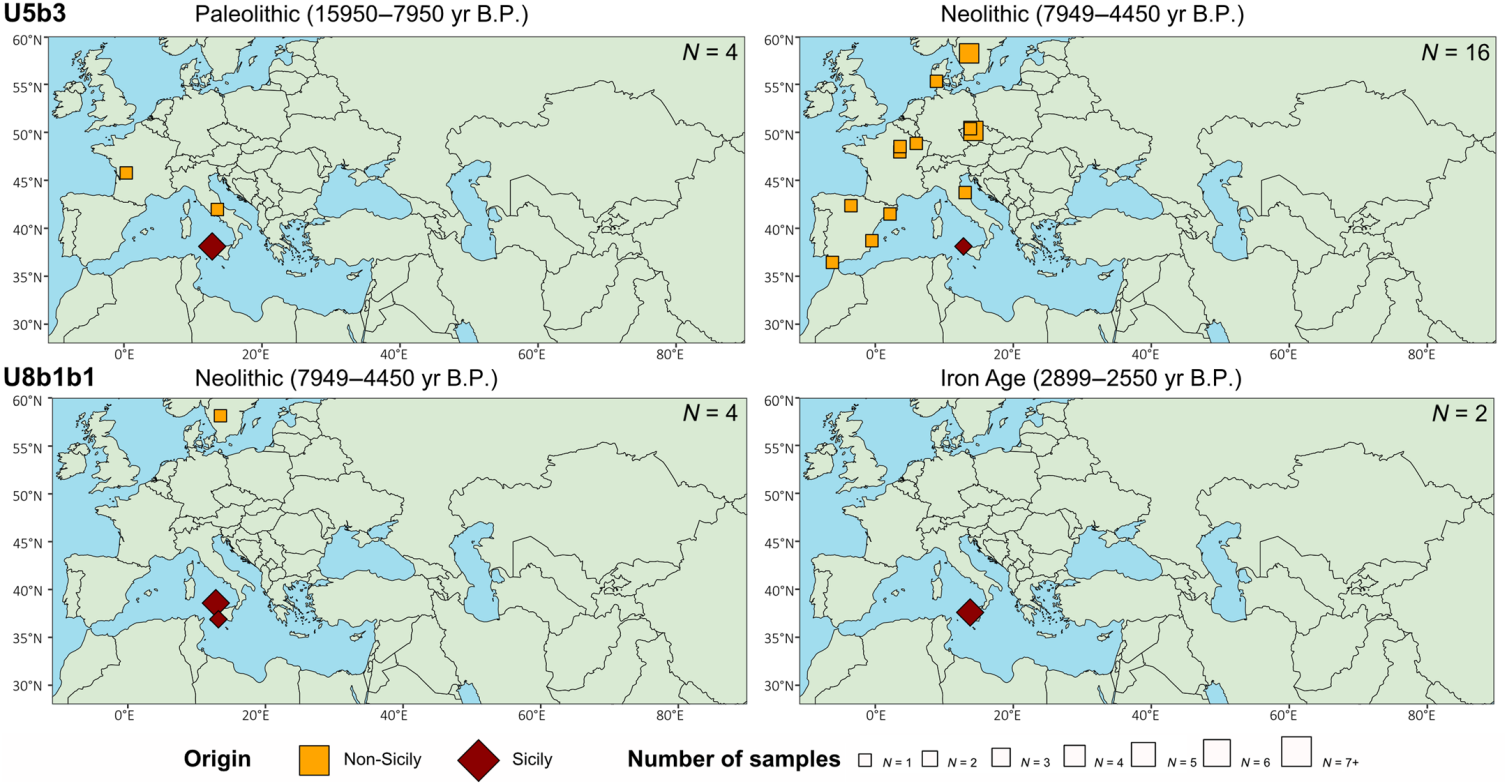
Archeological sites of U5b3 and U8b1b1 in different time transects, related to Sicily. Haplogroup U5b3 is dated to 17,822 yr B.P. (9809; 26,158 yr B.P.), while U8b1b1 is dated to 10,794 yr B.P. (6251; 15,456 yr B.P.). Orange squares represent non-Sicily sites, and brown diamonds Sicily sites (datasets S2 and S10B); their relative dimension is the number of samples per site. Total number of samples (of the related Hg) for each map in top right. See movie S1 for further details on these and other lineages (U5b1d1, U5b2b, K1a3, K1a4, and K2b1).

The U5b3 branch arose around 17,822 yr B.P. and was found in the Franco-Cantabrian region, in the Italian Peninsula, and in Grotta dell’Uzzo in Sicily (Fig. 4). This diffusion pattern suggests a clear exit from the Italian refuge, as previously demonstrated by Pala *et al.* 2009 (*26*). During the Neolithic period, it spread across western Europe, remaining present at Grotta dell’Uzzo. This pattern persists in the present day (0.64%) and extends to the rest of Europe and the Middle East.

### Neolithic signals

The lineage U8b1b1 first appeared in the European ancient dataset during the Neolithic period. Its occurrence was essentially confined to Sicily, with evidence found at Grotta dell’Uzzo and Contrada Buffa. The only additional finding is in a Neolithic family from Sweden (*34*). Subsequently, the lineage reappeared in the Iron Age only in Sicily at Polizzello, indicating that it likely survived on the island, where it is still present today despite its very rare frequencies across Western Eurasia (0.04%) (Fig. 4).

The K2b1 haplogroup emerged during the Neolithic period and was widespread in Britain, Central Europe, and Sicily. However, the Sicilian archeological site where it was identified (Fossato di Stretto Partanna) was culturally attributed to the Paleolithic period (*12*), which might indicate a correlation with the expansion of farming across an inner European route rather than through the Mediterranean Sea. During the Bronze Age, this haplogroup was present in Central Europe, Sardinia, and Sicily, specifically at Motya. In addition, it was identified in Sicily during the Iron Age at Polizzello and subsequently disseminated throughout Europe to the present day, with a current frequency of 0.22%.

The K1a3 branch is dated to 7373 yr B.P. and appears in Neolithic sites of Anatolia, while the Sicilian region was still in the Paleolithic period. The widespread distribution of K1a3 in the Near East and Europe since the Neolithic era provides compelling evidence that it is a mitochondrial marker for the spread of the agricultural revolution. The lineage manifested in Sicily only during the Bronze Age, at sites known as Marcita and Contrada Buffa, while it is completely absent in the modern era.

## DISCUSSION

This study presents a comprehensive analysis of mtDNA variation in Sicily spanning 15,000 years. The analysis is based on 236 modern mitogenomes and a dataset of 116 ancient mitogenomes (two newly generated). A statistically supported genetic discontinuity was identified in the Grotta dell’Uzzo dataset and dated between the Mesolithic and Neolithic periods across the Sicilian time transect of 15 millennia. After this transition, the best model accounts for genetic continuity in Sicily from the Neolithic to modern times. This contrasts with the discontinuity from the Bronze Age proposed by Fernandes *et al.* (*12*), suggesting instead a stable maternal gene pool that persisted over the millennia.

Our ancient mitochondrial dataset from Sicily revealed two distinct haplogroups, U5b and U8b/K, which differentially characterize the Paleolithic/Late Mesolithic and Neolithic periods, respectively. Detailed phylogenetic and demographic analyses focused on ancient and modern mitogenomes belonging to these two haplogroups confirmed a certain degree of population replacement in Sicily during the Mesolithic-Neolithic transition. This is the first instance in which this transition has been unambiguously confirmed by (matrilineal) mitogenome data in a microgeographic context. A decrease in the population size of U5b hunter-gatherers corresponded to a contemporary increase of early farming groups carrying U8b/K mitogenomes. This fluctuation may reflect a more complex scenario than a straightforward replacement of hunter-gatherers by Neolithic farmers. It may involve periods of population growth and decline, environmental changes, or rearrangements in social structures, which eventually affected the relative prominence of these groups in different periods of Sicilian history. Certainly, the slow diffusion of agriculture and cultivated plants by sea routes since about 9000 to 6000 yr B.P. gradually reduced the space available for hunting and gathering activities, which in turn triggered a steep population increase of the early farming communities. This is not new in the context of the Mediterranean, having already been identified in the Italian region of Calabria (*35*). A second slight decline of the hunter-gatherer–related haplogroups since approximately 4000/5000 yr B.P. may be attributed to additional migrations into the island, potentially linked to the spread of Indo-European pastoralists and to the more recent arrival of Phoenicians/Punics and Greeks.

Three different sublineages of U5b identified in ancient Sicilian mitogenomes have revealed a complex pattern of migrations following the LGM, as observed from the maternally inherited perspective offered by the mitogenome analysis. This mitochondrial pattern may be linked to different refuge areas, including the Italian peninsula (U5b3), the Balkans (U5b1d1), and Ukraine (U5b2b). On the other hand, different U8b/K sub-branches likely arrived in the island in consequence of different Neolithic waves, one (K1a3) across the Mediterranean Sea, while the other as an offset of inland European spread (K2b1). Moreover, we identified an additional and very rare Neolithic marker (U8b1b1) specific to the island of Sicily where it is quite common even in present day.

With regard to the present-day mtDNA gene pool of Sicily, no compelling evidence of genetic differentiation was detected among individuals currently living in different geogroups. These groups were delineated not only on the basis of political districts but also with due consideration for orographic barriers. This outcome challenges the east-west differentiation in Sicily, at least for mtDNA genetic variation, as inferred by analyses on genome-wide data (*7*): that east-west structure within Sicily only emerged at a refined level of hierarchical clustering (*k* = 14), and the two clusters appear closely related, showing some admixture within a genetically continuous area. Moreover, when we compared the eastern and western provinces analyzed by Sarno *et al.* (*7*) (Catania, Ragusa, Enna versus Palermo city/outer, Trapani, and Agrigento), we found significant differences also in our dataset (*P* value: 0.0004). The homogeneous genetic scenario observed in the more representative mtDNA dataset analyzed here likely reflects Sicily’s historical role as a key hub of Mediterranean trade and migration. The sole exception to this pattern is observed in Palermo city, where the high frequency of haplogroups of African origin stands out as a notable outlier. This could be attributed to recent migration across the Mediterranean Sea or within Sicily in modern times, although it may also be the result of more ancient migrations. Our ancient dataset indicates migration from Africa at different times (datasets S2), including the Bronze Age (one individual at the Motya archeological site) (*13*), the Iron Age (one individual at the Himera archeological site) (*36*), the Roman period (one in the Contrada Diana necropolis) (*11*), and the Middle Ages (two at the Segesta archeological site) (*37*). In the phylogenetic tree of Sicilian mitogenomes of African origin, it is evident that most of the contemporary mtDNAs are unique haplotypes directly descending from the ancestral node of the respective terminal haplogroup (21 of 36; dataset S11). Therefore, it was not possible to estimate any coalescence age for them. On the other hand, the Sicilian branches with different African origin haplotypes were dated with average estimates between 642 and 13,370 yr B.P. (fig. S5), which confirms the possibility of multiple migrations across the Mediterranean Sea or within Sicily.

In summary, the study of ancient and modern Sicilian mitogenomes provides robust evidence of a genetic continuity on the island following the initial genetic discontinuity between the Mesolithic and Neolithic periods. The dynamic interaction between hunter-gatherer and farmer groups adds complexity to the island’s demographic history, allowing us to identify specific mitochondrial sublineages of U5b and U8b/K able to mark different migrations from Western Eurasia that differentially shaped the mtDNA gene pool of Sicily in the Paleolithic and Neolithic periods, respectively.

The absence of significant genetic differentiation among modern mitogenomes further clarifies the genetic landscape of present-day Sicily, at least with regard to maternal lineages. The presence of African haplogroups in modern and ancient individuals, confirmed here through complete mitogenomes, highlights the island’s connections with Africa since the Bronze Age. These findings contribute to a deeper understanding of Sicily’s place in the broader narrative of Mediterranean and Italian prehistoric and historical migrations.

Our conclusions are based on a wide range of modern and ancient mitogenomes, but the latter are mainly from western Sicily. As a uniparentally inherited maternal locus, mtDNA only captures certain aspects of past demographic dynamics. A comparison with Y-chromosome data can help identify potential sex-biases in the population’s genetic history (*38*). Extending the survey to biparental markers may add and refine further past demographic processes and admixture layers (*7*). The strength of our study is in the use of high-resolution mitogenome diachronic data and a modern dataset that is widely representative of the island, which enabled fine-scale phylogeographic inference and provided valuable information for identifying migration patterns involving specific sublineages. Our inferences could be expanded by incorporating Y-chromosome data and conducting a broader analysis of whole genomes from modern and ancient individuals, including those from additional archeological sites in eastern Sicily. This broader analysis could capture additional aspects of past evolutionary and demographic dynamics involving Sicily and determine the extent to which these dynamics have influenced the genetic diversity of present-day populations.

## MATERIALS AND METHODS

### Experimental design

#### Modern sample collection

The modern collection consisted of 236 DNA samples from healthy and unrelated subjects with a Sicilian maternal grandmother as a terminal maternal ancestor (TMA; dataset S1). Buccal swab and mouthwash rinsing samples were collected from volunteers, representing the entire region of Sicily. Written informed consents were obtained from all donors, who provided information about place of birth and geographical origins, to select those with a Sicilian maternal grandmother as a TMA. DNA was extracted using the Maxwell Blood Kit automated extraction protocol, deemed suitable also for saliva samples.

#### Ancient DNA samples collection

The mitogenomes of the two ancient individuals excavated from Grotta dell’Uzzo in Trapani and Grotta di San Teodoro in Acquedolci (Messina) were assembled into a final dataset of 116 ancient mitogenomes from Sicily (dataset S2). This dataset includes publicly available data that can be used and published without restriction (*8*, *9*, *12*, *13*, *29*, *36*, *37*, *39*, *40*). They are from 16 different archeological sites, spanning from Paleolithic (including medium and late Mesolithic) to Medieval times. Sixty-six of them were produced and analyzed at the Laboratory of Molecular Anthropology and Paleogenetics (University of Florence). Thirty-nine complete mitochondrial genomes aligned to the human mitochondrial revised Cambridge Reference Sequence (rCRS; NC_012920.1) (*41*) in the BAM format were recovered from the European Nucleotide Archive (ENA; www.ebi.ac.uk/ena) (*33*), while 11 samples of Monnereau *et al.* (*37*) from Sequence Read Archive (SRA) (www.ncbi.nlm.nih.gov/sra) (*42*) in fastQ format.

#### Geographical and temporal division

Sicily was divided into nine subareas (geogroups), mostly based on the island’s provinces. We also took into account the heterogeneity of sample size and considered historical and orographic criteria. The genealogical information collected from the volunteers were analyzed to group the individuals based on the subarea place of birth of their TMA, as follows: Agrigento (*n* = 36), Caltanissetta-Enna (*n* = 21), Catania-Siracusa (*n* = 17), Gela (*n* = 21), Messina (*n* = 17), Palermo city (*n* = 65), Palermo outer (*n* = 27), Ragusa (*n* = 12), and Trapani (*n* = 20), thus representing the present-day demographic context of the Island (Fig. 1 and dataset S1).

We considered ancient samples in two ways by their temporal and geographical coordinates. They have been divided into six groups: Paleolithic, Neolithic, Bronze Age, Iron Age, Roman period, and Medieval times. Time periods were determined on the basis of the anthropological context of the archeological site and radiocarbon dating. We also considered them by geographical groups as we did with modern samples.

#### Ethical approval

All analyses were carried out in accordance with relevant guidelines and regulations, and all research protocols were approved by the Ethical Committee for Clinical Experimentation of the University of Perugia (protocol no. 2017-01 and 2020-94598) and by the Ethical Committee Fondazione IRCCS Policlinico San Matteo (protocol number 0028298/22). Written informed consents were obtained from all donors, who also provided information about place of birth and genealogy.

### Method details

#### Modern mitogenome sequencing

Complete mitogenome sequences were obtained through next-generation sequencing using an Illumina MiSeq system. Libraries were prepared following the Illumina DNA Prep protocol v10. After quality control with FastQC (*43*), FASTQ files were trimmed with Trim_galore v0.6.4_dev (*44*) to remove the adapters and the bases at the ends of each read with a Phred quality score lower than 30. Short reads were aligned to the human mitochondrial reference rCRS using Burrows-Wheeler Aligner (BWA) (*45*) with the specific *mem* algorithm, and then the BAM files were filtered and sorted with SAMtools (*46*). Duplicates were removed using Picard’s MarkDuplicates (*47*), and BAM files were downscaled according to their average depth with VariantBam tool (*48*). The variants were called using Mutect2 implemented in the Genome Analysis Tool Kit (GATK) (*49*) excluding regions prone to misalignment problems and mutation hot spots (applying mitochondria-mode option). Variants with less than 15% of frequency in our samples were removed with FilterMutectCalls (GATK package) and further filtered using BCFtools (*46*) to split multiallelic sites. Bcftools +setGT option was used to recognize heteroplasmies (mutations with frequency between 15 and 85%) and homozygous sites (one allele with frequency > 85%). Consensus sequence was then obtained using BCFtools. The final haplotypes were determined using Haplogrep 3.2.1 (*30*). Haplocheck (*50*) software has been used to check for the presence of contamination. Using Haplogrep report, we searched manually in original BAM files if missing marker sites (of a specific haplogroup) were present in our reads, and then we selected our sequences depending on quality criteria (no contamination, average depth > 10, genome coverage > 90%, and heteroplasmies number < 3).

#### Ancient mitogenome sequencing

Molecular analysis of the archeological specimens was performed under sterile conditions in a dedicated ancient DNA facility, at the Laboratory of Molecular Anthropology and Paleogenetics (University of Florence), following strict guidelines and standard precautions to avoid contaminations. Blank controls were processed in parallel with samples to monitor the contamination of the reagents. The outer layer of the tooth root was washed with a 2% sodium hypochlorite solution to remove surface contamination, rinsed with 95% ethanol to remove bleach, and irradiated by ultraviolet light (254 nm) for 10 min. For each tooth, ~50 mg of dentine powder recovered from the inner part of the root was used for DNA extraction, and the remaining amount of the root was used for radiocarbon dating. The details of extraction, library construction, target enrichment for mitochondrial genome, and sequencing are described in (*13*).

After demultiplexing, raw reads were analyzed using a specific pipeline developed for ancient DNA. AdapterRemoval (*51*) was used for initial sequencing quality control, adapter trimming, and paired-end read merging. Merged reads were filtered for a minimum length of 30 base pairs and mapped to rCRS using CircularMapper (*52*) (BWA parameters: -n 0.01, -l 16,500, -o 2). Only reads with a map quality score ≥ 30 were retained with SAMTools, and then polymerase chain reaction duplicates were removed with DeDup v.1.01 (*52*). MapDamage v2.0 (*53*) has been subsequently used to check and confirm the authenticity of the ancient DNA data. Samples not or partially UDG (Uracyl DNA Glycosylase) treated were soft clipped using trimBam of bamUtil v1.0.14 (*54*) (8 or 2 bases removed, respectively). The variant calling process was performed using the same tools as in the modern samples pipeline, but a different approach was used in determining which mutations should be retained during the filtering steps (minimum allelic fraction = 0.01, depth > 5, allelic frequency > 50%). Heteroplasmies were not considered. Consensus sequences, final haplotype, contamination control, and sequences selection have been performed as in the “Modern mitogenome sequencing,” except for heteroplasmies.

#### Comparative datasets and maps

We assembled the comparison dataset S10A by selecting studies on West Eurasia (defined as countries with longitudes between −15 and 60 and latitudes higher than 22) available on International Genome Sample Resource (IGSR) (*31*) by extracting mitogenomes from the CRAM files and then following the procedure outlined in the “Modern mitogenome sequencing” section; while for GenBank (*55*), we retrieved FASTA files and classified them using Haplogrep 3.2.1 (*30*).

We composed the dataset S10B by taking haplogroup classification and sample metadata directly from Allen Ancient DNA Resource (v62.0) (*56*, *57*) and from supplementary tables of original publications (*34*, *58*). For both datasets, we kept only individuals classified into intermediate haplogroups K1a3, K1a4, K2b1, U5b1d1, U5b2b, U5b3, and U8b1b1 (see datasets S10, A and B). All maps in this study were generated in R (*59*) using the packages rnaturalearth (*60*), ggspatial (*61*), and tidyverse (*62*).

#### Phylogenetic and statistical analyses

We grouped our individuals according to their macrohaplogroup and geographical origin. CA was performed using the package FactoMineR (*63*) in R (*59*). Chi-square test on the contingency table of the distribution of haplogroups was performed also using R to test for population structure. After removing all positions containing gaps and ambiguous data, an MP tree was built with mtPhyl v.5.003 (*64*) and then manually corrected using Haplogrep 2.4 (*65*) phylogenetic trees. Coalescence ages of haplogroups and subhaplogroups were calculated on the trees by means of the average number of base substitutions (rho-ρ) in the mtDNA, disregarding “indels,” hot spot transition at nucleotide position (np)16519 and variants around np309 (from np303 to np315) and np16189 (from np16182 to np16194). Standard error (sigma-σ) was calculated from an estimate of the genealogy (*66*). We adopted a newly proposed recalibrated-rho dating method by adding to the total number of mutations identified in the phylogenetic tree those that would have theoretically accumulated in each ancient sample based on its radiocarbon age. Subsequently, we converted the rho and sigma parameters into years as indicated by Soares *et al.* (*28*).

Further time estimates and demographic trends were evaluated using Bayesian Evolutionary Analysis of Sampling Trees (v2.7.7) (*67*). The Hasegawa-Kishino-Yano substitution model with gamma-distributed rate variation among sites (HKY + G) was selected as a common model for both datasets, as it has been widely adopted in human mtDNA studies, including Sardinia, the other major Mediterranean island (*14*), and other isolated populations (*68*).The transition/transversion ratio was estimated during the run, and the gamma distribution was discretized into eight categories to account for rate heterogeneity. A strict molecular clock was applied, assuming a constant substitution rate across all lineages. A coalescent Bayesian Skyline model was chosen as the tree before allow for fluctuations in past population size. The clock rate followed an informative normal prior with a mean of 2.45 × 10^−5^ and a SD of 3.5 × 10^−6^ (*28*). The radiocarbon ages of the ancient mitogenomes were incorporated into the Bayesian priors and modeled using a normal distribution in order to represent the 95% radiocarbon range reported in dataset S2. The Markov chain Monte Carlo chain was run for 10 million generations. Tracer v1.7.2 (*69*) was used to assess effective sample sizes and generate the BSP to visualize past demographic trends, together with the package ggplot2 (*70*) in R (*59*).

#### Population genetics analyses

To efficiently compare ancient and modern sequences, we performed a multiple sequence alignment with software MAFFT (*71*) with options FFT-NS-2 and-maxiterate 100. We calculated pairwise *F*_ST_ values (*19*) using the R package hierfstat (*72*) with the function genet.dist. We used the obtained *F*_ST_ values matrix to perform multidimensional scaling using the function cmdscale in the R environment.

#### Demographic modeling

To reconstruct the demographic and evolutionary dynamics in the island of Sicily, we simulated mitogenomes under alternative demographic models (fig. S2). We ran 50,000 simulations for each demographic model tested using fastsimcoal2 v.2.5.2 (*73*) within the ABCToolbox suite (*74*), which allowed us to sample ancient individuals at specific times and model continuous migrations through a migration matrix listing the migration rates as proportion of individuals moving from the source to the sink deme in each generation backward in time (dataset S4). Using arlsumstat v.3.5.2 (*75*), we computed the following summary statistics for each simulated dataset: within populations statistics: number of haplotypes (*K*), Heterozygosity (*H*), number of Segregating sites (*S*), Tajima’s D (*TAJIMAD*); between pairs of populations statistics: fixation index (*F*_ST_) and pairwise differences (*Pi*).

To identify the demographic model that best represents the observed genetic variation, we applied an ABC framework. Specifically, to perform the model selection analysis, we used an ABC approach based on a machine learning classifier, the random forest (RF) methodology (ABC-RF) (*76*). This machine learning optimization solves the “curse of dimensionality” arising from the use of a huge number of summary statistics and the need of millions of simulations per demographic model to produce a robust inference (*76*). Under this approach, the model selection procedure is rephrased as a classification problem, in which the machine learning algorithm first predicts the model that best fits the observed data with a Random-Forest classifier and then approximates its posterior probability. This RF classifier is trained on the simulated data using summary statistics as predictor variables and the demographic model that generated the statistics as the response variable. The summary statistics (specified above), computed for each demographic model compared, are used as a training dataset (reference table) for the ABC-RF analysis. We computed the same summary statistics in our observed data using arlsumstat v. 3.5.2 (*75*). In each comparison we used 500 trees. We computed the out-of-bag (*OOB*) prior error rate, which represents the prediction error, and a confusion matrix, which represents the accuracy of the classifier in distinguishing among the models tested, through the computation of *CE*s. In each model comparison, we performed a linear discriminant analysis (*LDA*) to investigate whether the simulated variation is able to capture the observed data. The model comparison analyses and relative statistics (*OOB*, *CE*, and *LDA*) were provided by the R package abcrf (*76*).

#### Models tested

We designed alternative demographic models following a hierarchical approach that consisted of three main comparisons. We considered 11 different groups of at least three individuals: Mesolithic Grotta dell’Uzzo (*n* = 8), Neolithic Grotta dell’Uzzo (*n* = 8), Chalcolithic Contrada Buffa (*n* = 4), Bronze Age Contrada Buffa (*n* = 3), Bronze Age Sicily (*n* = 14), Iron Age Sicily (*n* = 55), Phoenicians/Punics from Motya (*n* = 3), Greeks from Baucina (*n* = 3), Islamic individuals from Segesta (*n* = 5), Christian individuals from Segesta (*n* = 6), and all modern individuals (*n* = 236). In the first comparison, we considered five alternative demographic models (fig. S2, A to E) in which we sampled ancient populations at specific times based on sample dates (see model parameters and prior distributions, dataset S4). We tested a complete genetic continuity model (fig. S2A) in which all groups considered are in genetic continuity through time, while in the other models (fig. S2, B to E), we consider an initial separation between Sicilian populations and other Mediterranean populations, such as Phoenicians/Punics, Greeks, and Islamic individuals from Segesta. Each model describes hypotheses of genetic continuity and discontinuity through time, model B represents genetic continuity between Sicilian populations, model C accounts for a discontinuity in between Mesolithic and Neolithic populations, and models D and E describe genetic discontinuity from the Bronze Age and Iron Age respectively. In the second comparison, we investigate the effect of migration; starting from models B, C, D, and E (fig. S2), we account for continuous migrations (dataset S4) between Iron Age Sicily, Phoenicians/Punics, and Greeks during the Iron Age period and between Christian and Islamic individuals from Segesta during the Medieval period (fig. S2, F to I). As a third step, we directly compared the two most supported models identified in the previous model comparisons (respectively model A and model G).

Our last model comparison focuses on investigating a possible genetic structure between modern Sicilian populations. Starting from the best selected topology (model G), we designed two different demographic scenarios considering the modern Sicily population structured as the nine previously defined geogroups. The first model (model Gpan) describes a panmictic population in which all modern geogroups exchange migrants at high migration rate. In the second model (Model Gst), we considered east (Palermo city, Palermo outer, Trapani, and Agrigento) and west (Caltanissetta-Enna, Catania-Siracusa, Gela, Messina, and Ragusa) geogroups as two genetically distinct demes (fig. S4).

## Supplementary Materials

### The PDF file includes:

Provenance of ancient individuals Figs. S1 to S5 Legend for movie S1 Legends for datasets S1 to S11

### Other Supplementary Material for this manuscript includes the following:

Movie S1

Datasets S1 to S11
