# Supplementary material for: Fifteen millennia of human mitogenome evolution in Sicily
Source: Sci Adv. 2025 Nov 14;11(46):eady1674. doi: 10.1126/sciadv.ady1674 (PMC12617465; doi:10.1126/sciadv.ady1674)
Supplement: Supplementary file 1 — Provenance of ancient individuals Figs. S1 to S5 Legend for movie S1 Legends for datasets S1 to S11 [file sciadv.ady1674_sm.pdf]

Science Advances
AAAS

Supplementary Materials for

Fifteen millennia of human mitogenome evolution in Sicily

Anna Tommasi *et al.*

Corresponding author: Alessandro Achilli, alessandro.achilli@unipv.it; Silvia Ghirotto, silvia.ghirotto@unife.it

Sci. Adv. **11**, eady1674 (2025)
DOI: 10.1126/sciadv.ady1674

The PDF file includes:

Provenance of ancient individuals
Figs. S1 to S5
Legend for movie S1
Legends for datasets S1 to S11

Other Supplementary Material for this manuscript includes the following:

Movie S1
Datasets S1 to S11

Provenance of ancient individuals

We produced molecular data from the teeth of two ancient individuals excavated at two different archaeological sites in Sicily under the supervision of Prof. Luca Sineo (Dataset S2). The first tooth was entirely used for the molecular analyses and was excavated from Grotta di San Teodoro in Acquedolci (Messina) in 2021. This excavation was directly supervised by Prof. Sineo and approved by "Parco Archeologico di Tindari", Messina. The tooth was radiocarbon dated to 1993-1830 years BP at the facility for radiocarbon dating CEDAD (Centro di Fisica applicata, Datazione e Diagnostica, Università del Salento, Italy). The second tooth came from an individual, who was excavated from Grotta dell'Uzzo (Trapani) as part of the Agenda 2000 project (Prof. Sebastiano Tusa). This sample was radiocarbon dated to 8605-8430 years BP at CEDAD and is currently on display at the "Museo Archeologico Salinas" in Palermo, Italy.

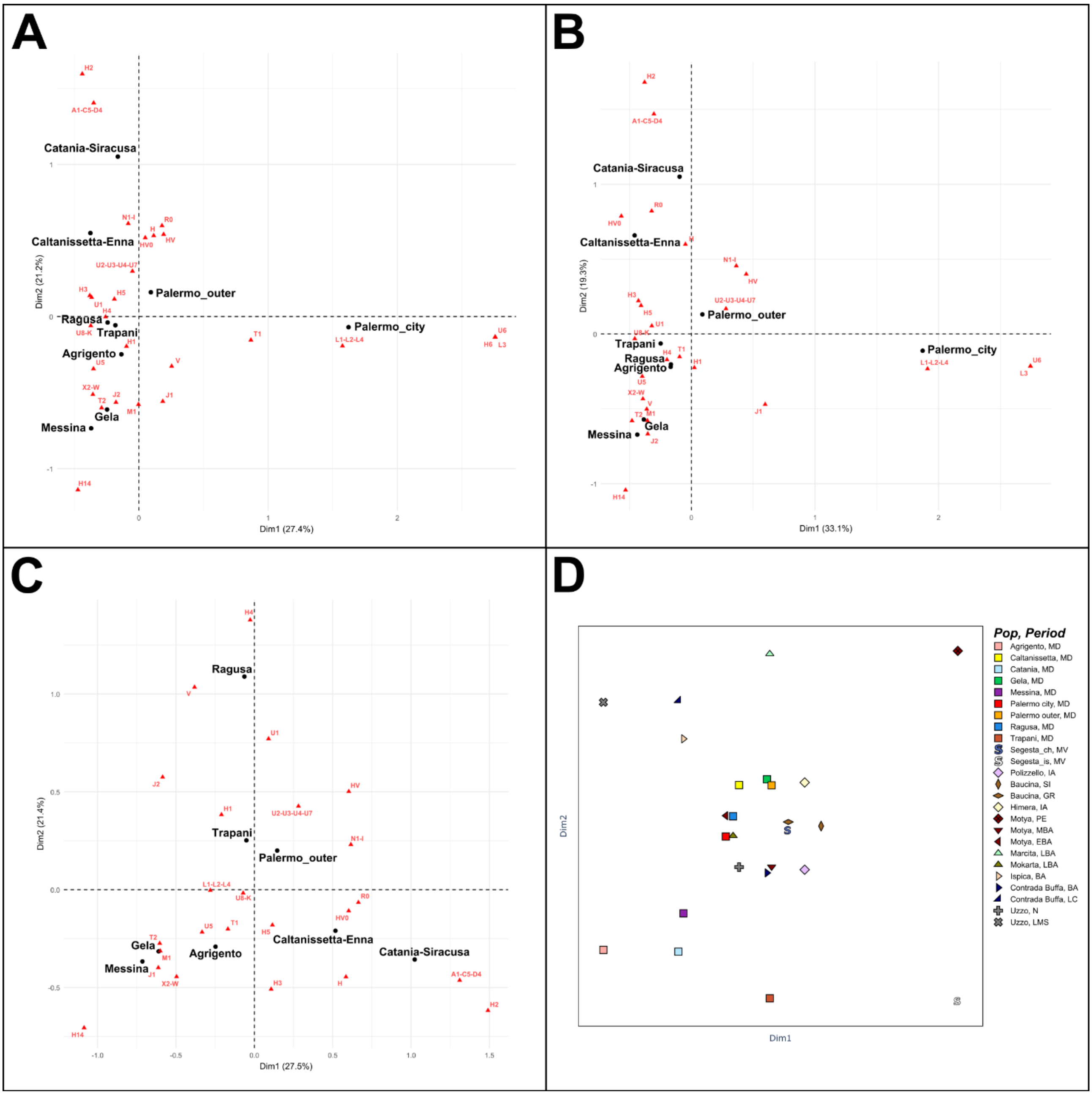

Fig. S1. Correspondence analyses and Multidimensional Scaling to investigate the presence of genetic structure. A) Correspondence analysis (CA) based on modern mtDNA haplogroup frequencies (27 macro-haplogroups, Dataset S1) in the eight established "geogroups". **B)** CA with Palermo with N=30. **C)** CA without Palermo. **D)** MDS based on Fst matrix from Main text Fig. 2.

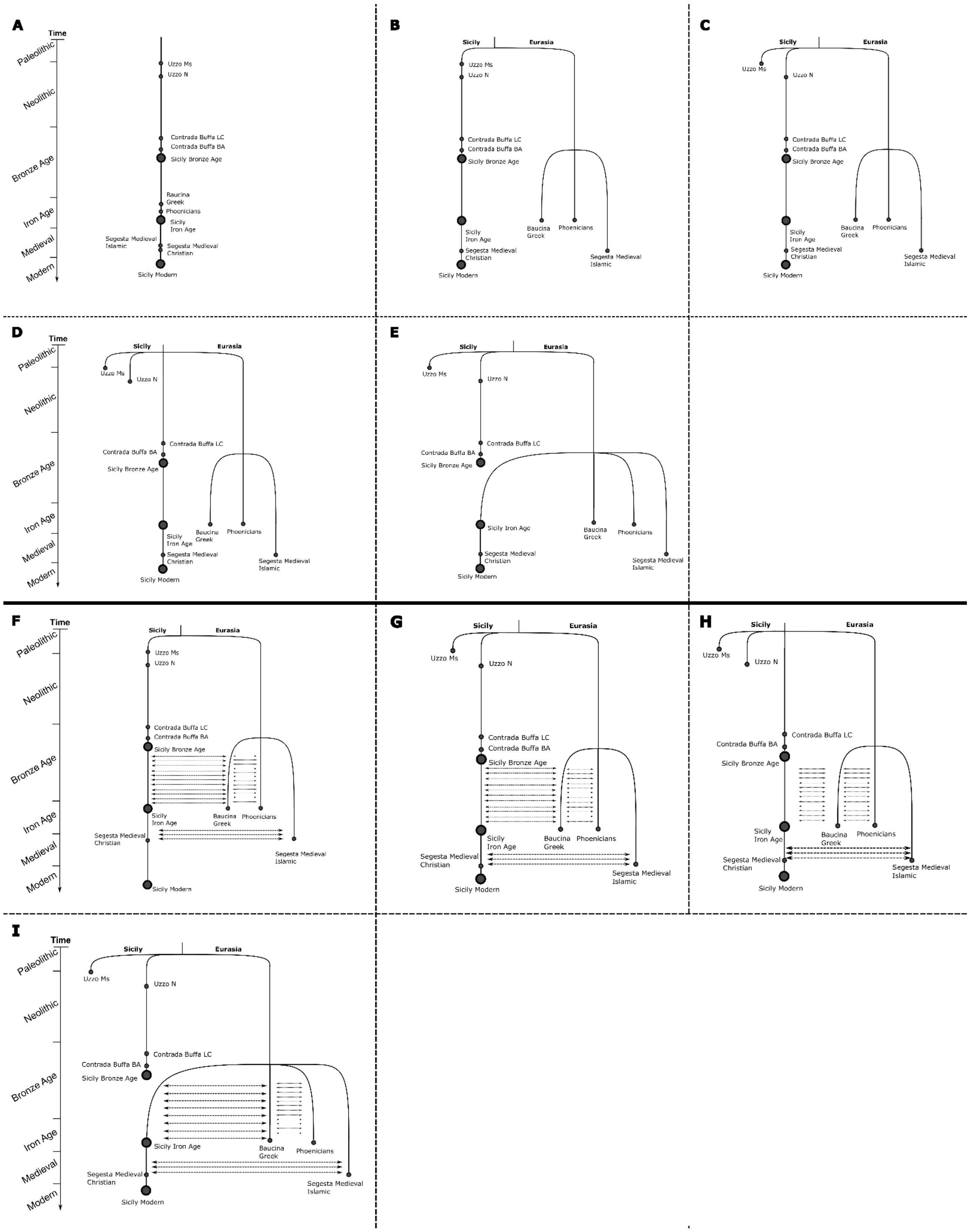

Fig. S2. Demographic models tested: models with no migration (A, B, C, D, E) and models accounting for migration (F, G, H, I). Continuous migrations are indicated by dotted arrows.

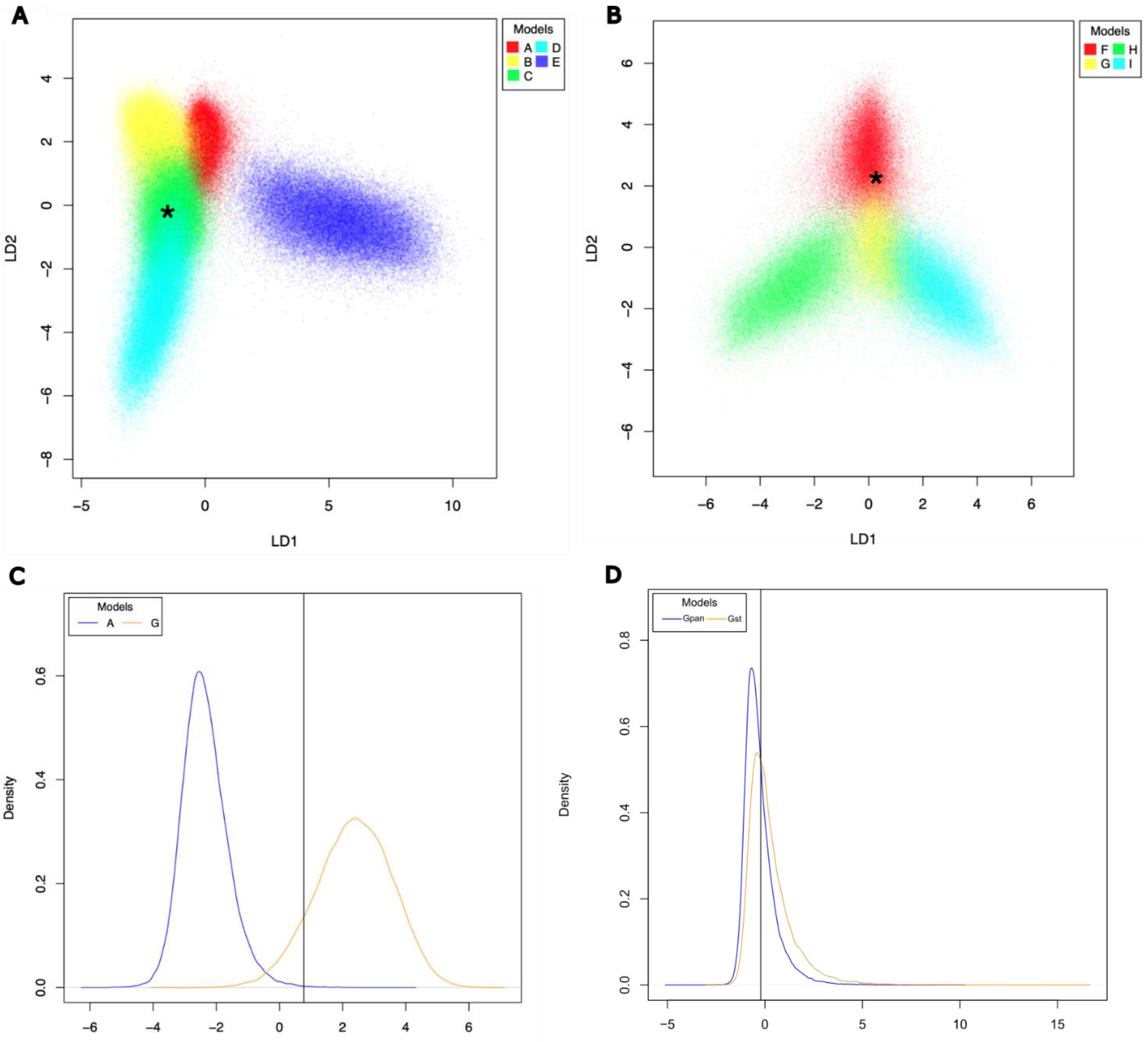

Fig. S3. Linear discriminant analyses for each model comparison. A) models with no migration. **B)** migration models. **C)** model comparison between models A and G. **D)** modern structure models (Gpan and Gst). Observed data are represented by a black star (A, B) or a vertical line (C, D).

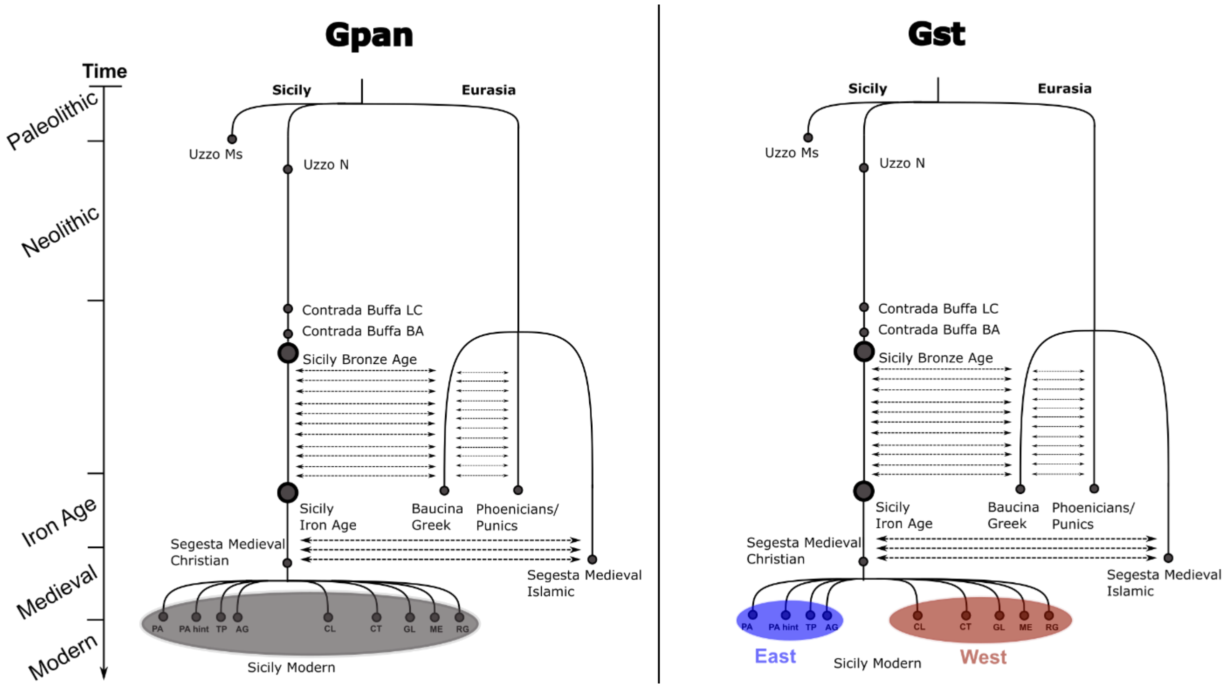

Fig. S4. Demographic models tested to investigate the presence of genetic structure between modern populations. A panmictic population model (Gpan) and a two (East and West) genetically distinct populations model (Gst).

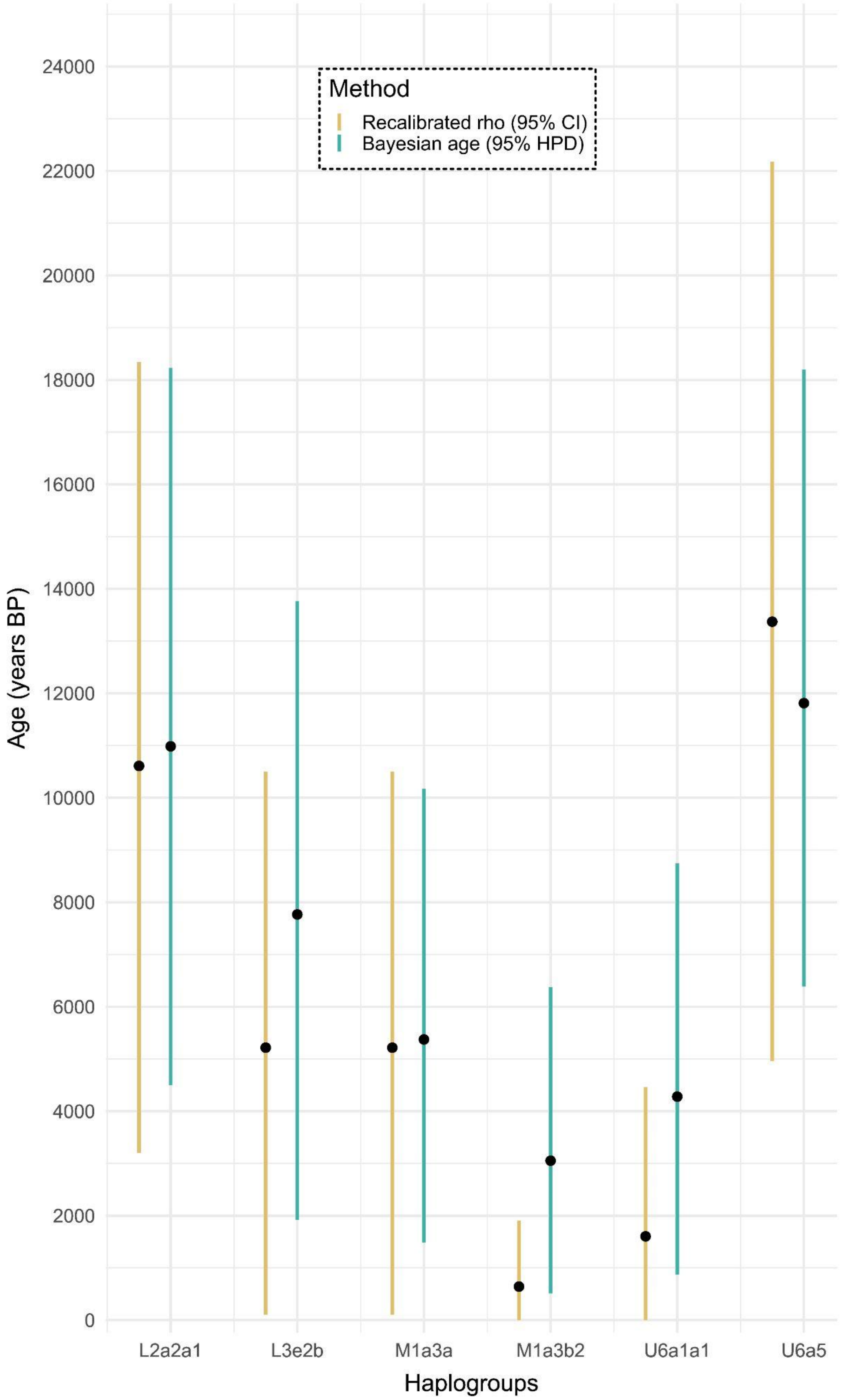

Fig. S5. Estimated ages of African lineages identified in Sicily. The X-axis shows the haplogroups, while the Y-axis lists the years before present. The golden line indicates the 95% confidence interval of the recalibrated ages, the blue line shows the 95% highest posterior density interval of the Bayesian estimated ages, and the black dots represent the mean values.

Movie S1. (separate file)
Distribution of haplogroups in Western Eurasia across time periods defined for Sicily, related to Figure 4.

Dataset S1. (separate file)
Complete mitogenomes of 236 modern Sicilians analyzed in this study.

Dataset S2. (separate file)
Dataset S2. Complete mitogenomes of 116 ancient Sicilians analyzed in this study. As for the downloaded data, if multiple sequence files are listed (separated by semicolons), they have been merged prior to analysis. Publicly available data could be used and published without any restriction.

Dataset S3A. (separate file)
Relative frequencies of macro-haplogroups across geogroups, for 236 modern Sicilians analyzed in this study. Absolute frequencies in brackets.

Dataset S3B. (separate file)

Relative frequencies of macro-haplogroups across archaeological sites, for 116 ancient Sicilians analyzed in this study. Absolute frequencies in brackets.

Dataset S4. (separate file)
Model parameters and prior distributions.

Dataset S5. (separate file)
Model selection results for each comparison in ABC-RF. Selected model in bold red.

Dataset S6. (separate file)
Model selection results for modern structure model comparison. Selected model in bold red.

Dataset S7. (separate file)
Age calculation for U8 and U5 branches using Soares et al. 2009. For further details see Materials and Methods.

Dataset S8. (separate file)
A putative most parsimonious tree of 15 complete mtDNA sequences from Sicilians under U5b branch. The tree encompasses 11 ancient and 4 modern Sicilian sequences. All sequences were compared with the revised Cambridge Reference Sequence (rCRS; NC_012920.1). Sequence gap positions were considered equivalent to the reference in the phylogenetic analysis. The coalescence times of haplogroups with at least three DNA sequences are indicated in the nodes. The first date is calculated by adding to the total

number of mutations those that would have accumulated in each ancient sample based on its radiocarbon age, while the second is considered based on all indicated substitutions. Mutations are transitions unless a base is explicitly indicated. Suffixes indicate reversions (@). Heteroplasmy (het) are highlighted in notes. Recurrent mutations are underlined. Insertions and deletions were disregarded. The geographic origin of each sample is indicated with specific colors; ancient samples are sage green shaded.

Dataset S9. (separate file)

A putative most parsimonious tree of 28 complete mtDNA sequences from Sicilians under the U8 branch. The tree encompasses 18 ancient and 10 modern Sicilian sequences. All sequences were compared with the revised Cambridge Reference Sequence (rCRS; NC_012920.1). Sequence gap positions were considered equivalent to the reference in the phylogenetic analysis. The coalescence times of haplogroups with at least three DNA sequences are indicated in the nodes. The first date is calculated by adding to the total number of mutations those that would have accumulated in each ancient sample based on its radiocarbon age, while the second is considered based on all indicated substitutions. Mutations are transitions unless a base is explicitly indicated. Suffixes indicate reversions (@). Heteroplasmy (het) are highlighted in notes. Recurrent mutations are underlined. Insertions and deletions were disregarded. The geographic origin of each sample is indicated with specific colors; ancient samples are sage green shaded.

Dataset S10A. (separate file)

Complete mitogenomes of 586 modern West Eurasians for selected haplogroups. Note that only the haplogroup classification of each sample was used for the analysis, but all mitogenomes were reclassified using Haplogrep 3.2.1. Publicly available data could be used and published without any restriction.

Dataset S10B. (separate file)

Dataset S10B. Complete mitogenomes of 352 ancient West Eurasians for selected haplogroups. Note that only the original haplogroup classification of each sample was used for the analysis. Publicly available data could be used and published without any restriction.

Dataset S11. (separate file)

A putative most parsimonious tree of 41 complete Sicilian mtDNA sequences of African origin.
